# THE DIAL-UP BLUES: OPINIONS ON TELE-HEALTH AND BROADBAND AMONG RURAL RESIDENTS

**DOI:** 10.1093/geroni/igz038.1300

**Published:** 2019-11-08

**Authors:** Aisha Bonner Cozad

**Affiliations:** AARP, Washington, District of Columbia, United States

## Abstract

Findings from several AARP telephone studies on Caregiving and telehealth will be presented. Starting in 2017, AARP began collecting data on telehealth issues in the caregiving studies. As the amount of data began to grow, patterns of telehealth use began to emerge. Using a sample of 1,000 respondent’s age 45-plus, the data shows respondents are generally supportive of the telehealth. Telehealth activities are often billed as a way to reduce healthcare cost and to provide much needed healthcare to geographically isolated populations particularly those residing in rural areas. A key component to accessing telehealth services is having access to adequate broadband which can be challenging in rural communities. Data shows that older adults (65+) are less likely to be very interested in telehealth services. Research finds that those employed, women and AARP members are all more likely to be very interested in using the telehealth services.

